# The Grand Challenge in Cranial Pain—From Migraine to Cranial Neuralgias: Understanding Differences and Similarities to Advance Knowledge and Management

**DOI:** 10.3389/fneur.2017.00019

**Published:** 2017-02-01

**Authors:** Cristina Tassorelli

**Affiliations:** ^1^Headache Science Centre, C. Mondino National Neurological Institute, Pavia, Italy; ^2^Department of Brain and Behavioral Sciences, University of Pavia, Pavia, Italy

**Keywords:** headache, primary headache, cranial neuralgias, challenges, migraine

The field of primary headaches and cranial neuralgias has experienced in a relatively brief period a startling progress in expansion of knowledge (1, 2). This has allowed partial unraveling of the machinery involved in the occurrence of attacks and the identification of a set of mediators and genes that are likely to play a role. In the case of migraine, accumulated evidence has very recently led to the development of the first disease-specific prophylactic drug, the CGRP antibodies (3). In other cases, drugs available for indications different from pain, such as botulinum toxin or the antiepileptic drugs, have been tested with adapted protocols proving effective in chronic migraine and showing potential efficacy in other cranial pains, i.e., cluster headache, trigeminal neuralgia, and temporomandibular joint disorder (4, 5).

Notwithstanding the undeniable scientific advances, much research is still needed to fully understand and effectively treat primary headaches and cranial neuralgias. Open issues are manifold, spanning from pathophysiology to clinical aspects, and represent exciting challenges for future research.

## The Challenge of Pathophysiological Components of Cranial Pain

### The Anatomy

The trigeminovascular system has been implicated as the major sensory system that mediates primary and secondary headaches of intracranial origin. More recent is the discovery that sensory innervation of the calvarial periosteum overlying the dorsal parts of the frontal and parietal calvarial bones is supplied primarily by trigeminal neurons with cell bodies located mainly within the ophthalmic division of the trigeminal ganglion (6). Taken together with the limited knowledge about the functional anatomy of the neurovascular unit, these data call for more investigations aimed at unraveling the functional anatomy and the physiology of cranial pain.

### The Genes

Genetic studies have contributed significantly to the current understanding of the molecular pathophysiology of hemiplegic migraine as a channelopathy. Beyond and besides channelopathies, several genetic variants, regulating different functions, with higher frequency and lower effect have been implicated in the most common forms of migraine (7). Evidence continues to accumulate from association studies for the involvement of the originally suspected vascular system dysregulation, for a modified inflammatory state, and for the susceptibility to migraine lying at the intersection of increased ascending nociceptive signaling and reduced descending inhibition (8). Migraine stands at the cross road of these functional pathways, with a genetic variability that is consistent with the well-known heterogeneous nature of this disorder.

### The Receptors

Identification and cloning of nociceptive receptors has tremendously advanced our understanding of the biology of nociception and multimodal pain sensation (9). In-depth characterization of functional properties of these receptors, as well as their variable expression in different tissues and cell types within the nervous system, will prompt the possibility to transfer acquired knowledge into clinically relevant advances.

### The Ictal Nature

Most primary headaches, in analogy to cranial neuralgias, are chronic conditions, recurring for years/decades, but manifesting with episodic flares during which normally functioning individuals suddenly become very sick and disabled. It is of course paramount to keep investigating the mechanisms that trigger the sickness, but it seems even more important to decipher the self-healing mechanisms. Indeed, the capability to recover functional integrity after a variable amount of time (minutes, hours, or a few days) points to the existence of endogenous mechanisms that are highly effective in reinstating homeostasis, to suggest that the machinery for controlling the disorder is intrinsically preserved in these patients, just temporarily disrupted. Focusing our efforts on the clarification and the potentiation of the mechanisms that restore normality will prompt additional therapeutic targets and options for treating these disorders, in addition to striving to prevent the initiation of the attack.

Unveiling these aspects will not only improve understanding and management of episodic headache and neuralgia, but it will also greatly benefit the prevention of those headaches that, episodic in nature, are at higher risk of progressively becoming chronic (10). This is mostly observed in some types of primary headache, but the phenomenology does not spare secondary headaches (i.e., post-traumatic headache, cervicogenic headache, TMJ dysfunction) and neuralgias.

## The Challenge of Sex Differences

It is well established that the majority of primary headaches and other trigeminal nerve-associated disorders have higher prevalence in females than in males (11, 12). Migraine has a twofold to threefold increase in prevalence in women as compared to men, and women experience longer duration of attacks, increased risk of attack recurrence, greater disability, and a longer period of time required to recover (11). There is evidence implicating the role of female sex hormones as a major factor in determining migraine risk and characteristics, which accounts for sex differences: emerging data suggest a sex-driven differential activation of inflammatory pathways (13, 14) and support underlying genetic variance of specific genes, i.e., estrogen receptor 1 gene (8).

Besides biological factors, psychosocial processes, such as pain coping and early-life exposure to stressful events, may also explain sex differences in pain (15). Therefore, the future directions of research should aim at further elucidating mechanisms that may inform efforts to develop sex-specific treatments. In this frame, an additional area of interest is represented by the mechanisms that influence age-related changes in gender segregation of some painful conditions of the head.

## The Challenge of Behavioral Aspects

Psychobiological events, together with cognitive modulation and reward processing have been implicated in chronic orofacial pain, including chronic migraine, trigeminal neuropathic pain, and pain related to temporomandibular joint disorders (16, 17). Scientific knowledge is quite rudimental in this regard, and more attention should be focused on the behavioral aspects of pain and pain management. Advances in this field will not only provide an important improvement in the understanding of headache pain but also prompt more specific therapeutic modalities.

## The Challenges in the Clinical Field

In the clinical field, challenges involve the need to build more evidence in *ad hoc* controlled studies to prompt the possibility to create and implement specific guidelines for the diagnosis and management of headaches and head pain. A paradigmatic case is represented by medication overuse headache, a secondary headache usually presenting in migraineurs in association with the excessive use of acute drugs. The condition is treatable but an evidence-based consensus on the management is missing. Is detoxification from overused drugs necessary? Is the association of detoxification plus prophylactic treatment more effective than either modality alone? Another example is represented by the nosographic framing of trigeminal neuralgia, which is undergoing dynamic shaping among experts (18, 19).

Evidence-based data are also needed to optimize treatment in terms of selection of drugs with the best efficacy/tolerability profile for a specific patient, to define duration of prophylactic cycles, and to identify subpopulations at risk of disease progression toward a negative outcome.

Finally, but not less important, a challenge that is transversal to all chronic disorders is represented by the development and implementation of informatic tools and services aimed at facilitating the remote management of these patients, which is likely to improve the quality of care and the outcome of disease (20).

## The Challenge of Differences That Enlighten

Finally, but no less importantly, because in itself it justifies the existence of this section of *Frontiers in Neurology*, the utmost challenge is the clarification of similarities and differences between migraine and cranial neuropathic pain. Their pathophysiological backgrounds involve several similar mechanisms (21). Peripheral sensitization occurs in neurons located in the dorsal root ganglion or in the trigeminal ganglion, while central sensitization appears in the second-order neurons in the dorsal horn of the spinal cord or the trigeminal nucleus caudalis. Central neuronal hyperexcitability has been implicated in both disorders, as are alterations in the glutamatergic neurotransmission and activation of *N*-methyl-d-aspartate-receptors, according to increasing evidence. Furthermore, migraine and neuropathic pain share some clinical features, such as enhanced sensitivity to sensory stimuli and cutaneous allodynia. The pharmacotherapy of both diseases is often challenging, but both respond to antiepileptic drugs (22). Building on these similarities, and unraveling the specific differences, might prompt novel candidates for the development of future drugs, hopefully more efficacious. The process has already started with the metabolites of the kynurenine pathway, for their capability of influencing the glutamate receptors (23).

## Author Contributions

The author confirms being the sole contributor of this work and approved it for publication.

## Conflict of Interest Statement

The author declares that the research was conducted in the absence of any commercial or financial relationships that could be construed as a potential conflict of interest.
